# IL-22 promotes tumor growth of breast cancer cells in mice

**DOI:** 10.18632/aging.103439

**Published:** 2020-07-10

**Authors:** Ying Zhang, Cong Liu, Jun Gao, Siqi Shao, Yingying Cui, Songlou Yin, Bin Pan

**Affiliations:** 1 Department of Pathology, Laboratory of Clinical and Experimental Pathology, Xuzhou Medical University, Xuzhou 221004, China; 2 Department of Hematology, The Affiliated Hospital of Xuzhou Medical University, Xuzhou Medical University, Xuzhou 221002, China; 3 Blood Diseases Institute, Xuzhou Medical University, Xuzhou 221002, China; 4 Department of Rheumatology, The Affiliated Hospital of Xuzhou Medical University, Xuzhou Medical University, Xuzhou 221002, China

**Keywords:** breast cancer, interleukin-22, innate lymphoid cell group 3

## Abstract

Increased interleukin-22 (IL-22) level was reported to associate with progression of breast cancer. Regulation of IL-22 in breast cancer still needs to be elucidated. We assessed the effect of giving IL-22 in tumor growth of mice inoculated with 4T1, MCF7 and MDA-MB-231 breast cancer cells. IL-22-producing cells were analyzed in tumor tissues. We also analyzed the impact of giving IL-1β and IL-23 on IL-22 levels in tumor tissues. Giving exogenous IL-22 increased tumor size and intra-tumor Ki-67-positive cells *in vivo*. IL-22 increased phosphorylated STAT3 level and proliferation of breast cancer cells *in vitro*, an effect blocked by a STAT3-inhibitor stattic. Endogenous IL-22 mRNA level was up-regulated in tumor tissue, compared with normal mammary tissue. Innate lymphoid cell group 3 (ILC3) is a major producer of IL-22 in 4T1 tumor. Giving IL-1β and/or IL-23 increased cell proliferation in 4T1 tumor, which was reversed by concurrent use of an IL-22 neutralization antibody. IL-1β and IL-23 increased levels of IL-22 mRNA and IL-22-producing ILC3 in 4T1 tumor. Our findings suggest a mechanism for how IL-22 regulates tumor growth in breast cancer, and indicate blocking IL-22 function might reduce IL-1β- and IL-23-induced tumor progression of breast cancer.

## INTRODUCTION

Breast cancer is the most common malignancy in women, causing significant morbidity and mortality. Progress in surgical treatment and adjuvant systemic therapies significantly improve survival of patients [1]. However, some cases are resistant to adjuvant therapies and therefore remain incurable [2]. Mechanisms mediating pathogenesis of breast cancer still need to be elucidated.

Tumor microenvironment, particularly in those with advanced stage diseases, plays an important role in supporting tumor growth, mediating drug resistance and promoting metastasis [3]. There is considerable interest in exploring the role of immune compartment of the tumor microenvironment, such as lymphocytes and innate immune cells which can be found in most solid tumors. These studies reported two main aspects of dysfunction regarding immune microenvironment: firstly, immune cell-derived growth factors promote tumor growth, such as vascular endothelial growth factor (VEGF) and epidermal growth factor (EGF); secondly, immunosuppressive effector molecules mediate immune escape of tumor cells, for example, transforming growth factor-β (TGF-β) and programmed death-ligand 1 (PD-L1) [4–6]. Thus, new strategies in controlling breast cancer progression had emerged accordingly.

Interleukin-22 (IL-22), an IL-10 family member, exerts an effect in regulating epithelial homeostasis [7]. IL-22 stimulates proliferation of epithelial cells in gut and thymus. We and others previously showed the primary cellular sources of IL-22 are innate lymphoid cell group 3 (ILC3), T-cell and macrophage [8, 9]. IL-22 shows an immune suppressive function in regulation of adaptive immune responses. The epithelial proliferative and immunosuppressive effects can be utilized by cancer cells to facilitate tumor growth [7, 10]. This hypothesis was proposed according to the association between IL-22 and several types of tumors [11–14]. Infiltration of ILC3 and increased IL-22 level were reported to correlate with progression of breast cancer [15, 16]. However, regulation of IL-22 in breast cancer still needs to be elucidated.

Based on these reports and our previous findings, we studied the role of IL-22 in tumor growth of breast cancer. We found IL-22 stimulated proliferation of breast cancer cells in a signal transducer and activator of transcription 3 (STAT3)-dependent manner. In 4T1 breast cancer model, ILC3 was a major producer of IL-22, which was increased by giving IL-1β and IL-23.

## RESULTS

### IL-22 promotes growth of breast cancer cells *in vivo*

**Figure 1:**
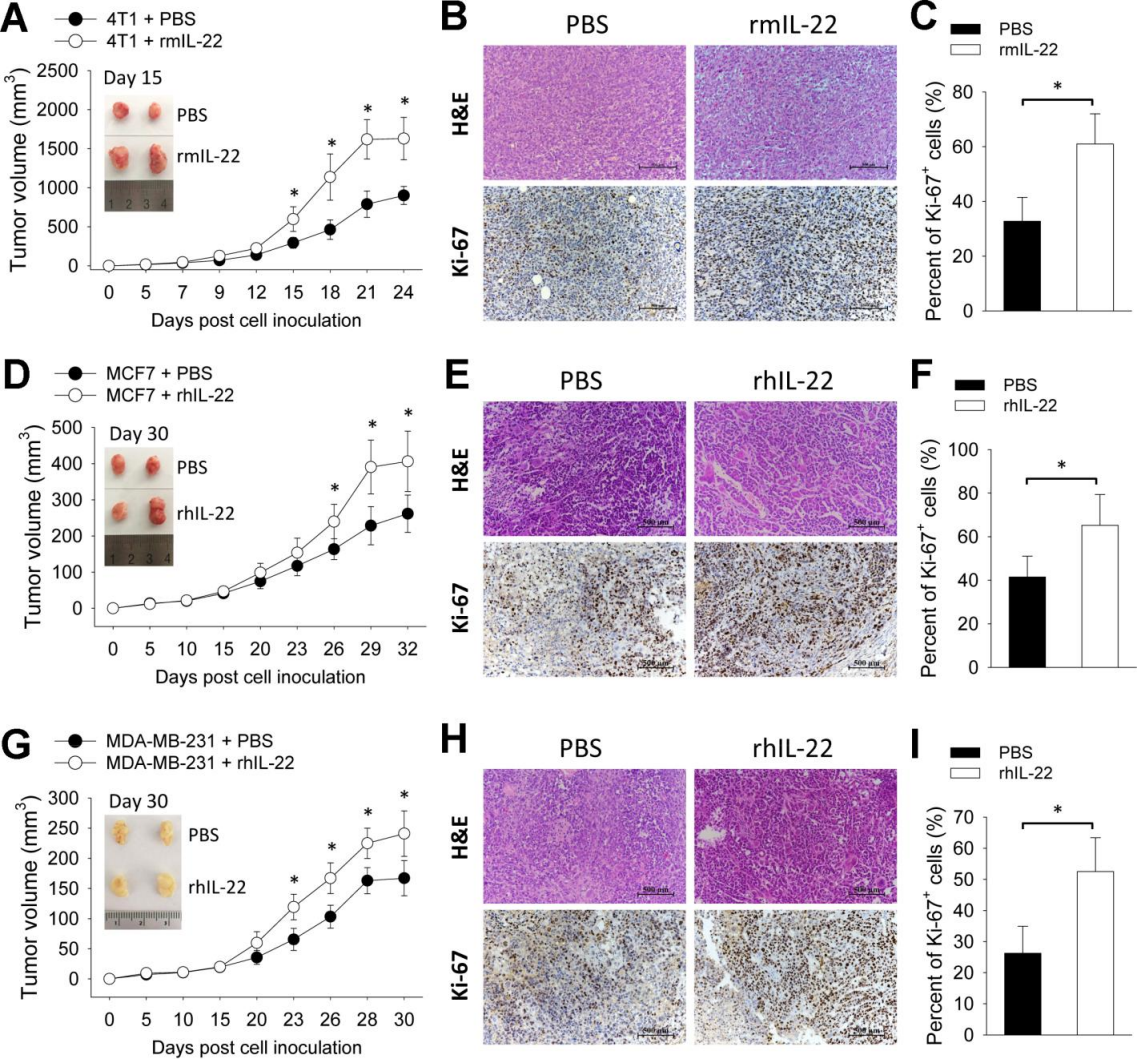
**IL-22 promotes growth of breast cancer cells *in vivo*.** (**A**–**C**) 4T1 cells (2×10^5^) were injected into a single mammary fat pad of BALB/c mice (n=6). (**D**–**F**) MCF7 cells (2×10^6^) and (**G**–**I**) MDA-MB-231 cells (2×10^6^) were inoculated subcutaneously to BALB/c Nude mice (n=6). From day 3 post cell inoculation, mice were injected with PBS or recombinant murine IL-22 (rmIL-22, 20 μg/kg) or recombinant human IL-22 (rhIL-22, 20 μg/kg) thrice weekly for up to 3 weeks. (**A**, **D**, **G**) Tumor size was measured continuously. (**B**, **E**, **H**) 4T1 tumors were collected on day 15. MCF7 and MDA-MB-231 tumors were collected on day 30. Histological analyses were performed by H&E staining and Ki-67 immunohistochemical staining. Scale bar: 500 μm. (**C**, **F**, **I**) Percent of Ki-67-positive cells were counted (n=4). Data are from two independent experiments. Data are presented as mean ± SD, compared using unpaired *t* test. *p < 0.05.

To assess the impact of exogenous IL-22 on tumor growth, we applied three breast cancer models by transplanting 4T1 cells to wild type BALB/c mice and transplanting MCF7 cells and MDA-MB-231 cells to BALB/c Nude mice. In the 4T1 model, injection of IL-22 increased tumor size (Figure 1A) which was associated with increased proliferation of tumor cells as indicated by more Ki-67-positive cells (Figure 1B, 1C). This effect was also observed in MCF7 and MDA-MB-231 xenograft models (Figure 1D–1F). These results show that IL-22 promotes growth of breast cancer cells *in vivo*.

### IL-22 stimulates proliferation of breast cancer cells *in vitro*

**Figure 2:**
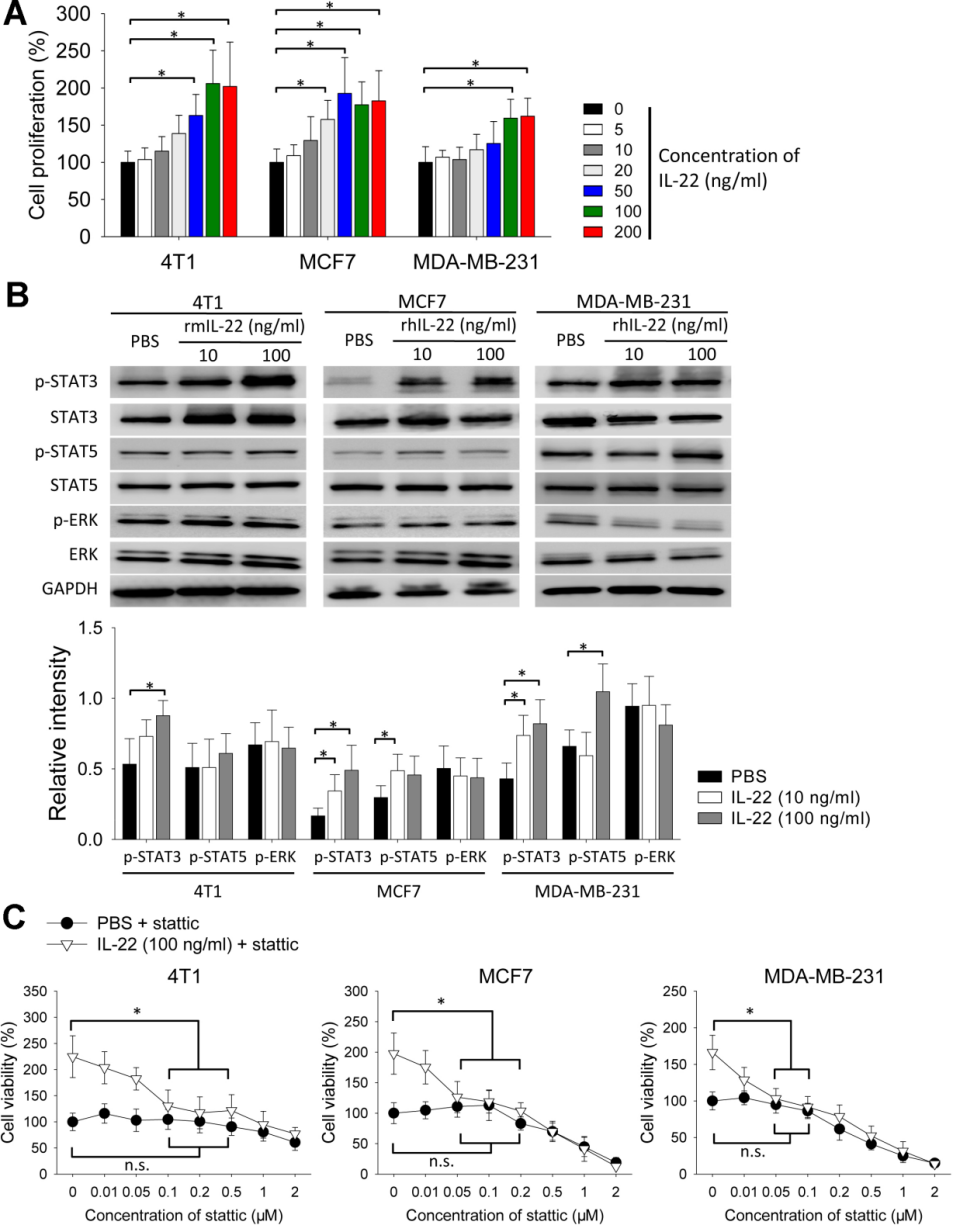
**IL-22 stimulates proliferation of breast cancer cells *in vitro*.** (**A**, **B**) 4T1, MCF7 and MDA-MB-231 cells were cultured with different doses of rhIL-22 or rmIL-22 for 24 h. Cells cultured without IL-22 were used as control. (**A**) Cell proliferation was measured with CCK-8 assay (n=6). *, *p* < 0.05. (**B**) Western blot was applied to analyze levels of indicated proteins. Experiment was repeated twice. Intensities of phospho-proteins were analyzed using ImageJ software, and were normalized to relative total proteins. *, *p* < 0.05. (**C**) 4T1, MCF7 and MDA-MB-231 cells were cultured with IL-22 (100 ng/ml) in the presence of different doses of stattic for 24 h. Cells cultured without stattic were used as control. Cell proliferation was measured with CCK-8 assay (n=6). *, *p* < 0.05, compared with control in IL-22 (100 ng/ml) + stattic group; n.s., not significant, compared with control in PBS + stattic group. Data are mean ± SD, compared using unpaired *t* test or one-way ANOVA test.

Next, we tested the effect of IL-22 in cell proliferation *in vitro*. IL-22 increased proliferation of 4T1, MCF7 and MDA-MB-231 cells (Figure 2A). Because IL-22 signal is transduced by STAT and extracellular signal-regulated kinase (ERK) pathways [7], we detected phosphorylation of these proteins. IL-22 increased levels of phosphorylated STAT3 in 4T1, MCF7 and MDA-MB-231 cells. IL-22 also increased phosphorylation of STAT5 in MCF7 and MDA-MB-231 cells but not in 4T1 cells. In contrast, IL-22 did not increase phosphorylation of ERK in these cells (Figure 2B). To test whether STAT3 is important for IL-22 mediated proliferative effect, we added STAT3-inhibitor stattic to cell cultures. IL-22-induced cell proliferation was reversed by stattic at the concentration ranging from 50 to 500 nM. In contrast, the same dose of stattic did not decrease proliferation of cells which were not treated with IL-22 (Figure 2C). These findings indicate IL-22 stimulates proliferation of breast cancer cells in a STAT3-dependent manner.

### Cellular source of IL-22 in breast cancer

**Figure 3:**
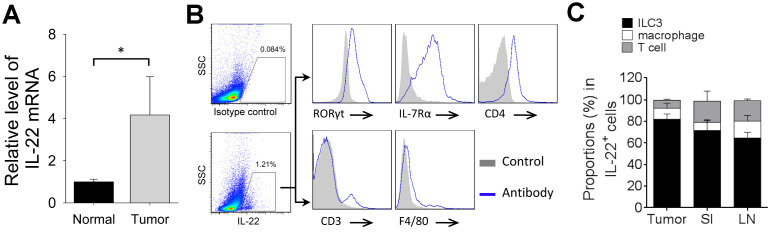
**Cellular source of IL-22 in 4T1 tumor.** (**A**) Total RNAs were extracted from tumor tissue or normal mammary tissue in 4T1 tumor model. qPCR was applied to analyze levels of IL-22 mRNA (n=4). Data represent fold changes. (**B**, **C**) Tumors, small intestine and mesenteric lymph nodes were collected from mice on day 21 after cell injection. Flow cytometry was applied to analyze IL-22-producing cells. (**B**) Figure shows cells gated from IL-22-positive population and expression of indicated markers. T cells are defined as CD3^+^. Macrophages are defined as F4/80^+^. ILC3s are defined as CD3^-^CD4^+^IL-7Rα^+^RORγt^+^. (**C**) Proportions of ILC3, macrophage and T-cell in IL-22-positive cells (n=4). Data are mean ± SD, compared using unpaired *t* test. *, *p* < 0.05.

Next, we analyzed the endogenous IL-22 in 4T1 tumor model. Tumor tissue expressed higher level of IL-22 mRNA than that in normal mammary tissue (Figure 3A). IL-22-producing cells were detected in tumor tissue. Most IL-22-positive cells were ILC3s, whereas T-cell and macrophage took smaller proportions in IL-22-positive cells (Figure 3B, 3C). We also analyzed IL-22-producing cells in small intestine and lymph node. Percent of IL-22-positive ILC3 in tumor tissue was higher than that in lymph node (*p* < 0.05). Percent of IL-22-positive macrophage in lymph node was higher than that in small intestine (*p* < 0.05). Percent of IL-22-positive T-cell in small intestine and lymph node were higher than that in tumor tissue (*p* < 0.05) (Figure 3C). Thus, ILC3 is a predominant producer of IL-22 in tumor tissue of 4T1 model.

### IL-1β and IL-23 increases intro-tumor level of IL-22 and promotes growth of breast cancer cells

**Figure 4:**
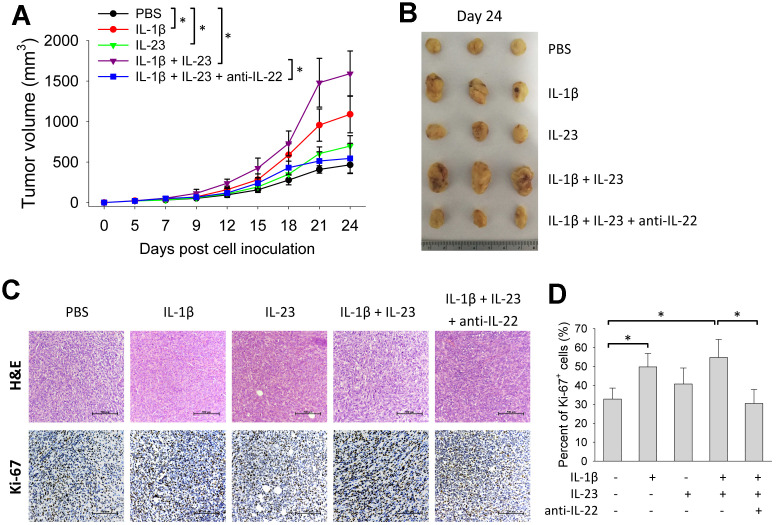
**IL-1β and IL-23 promotes growth of 4T1 tumor.** BALB/c mice (n=6) were injected with 4T1 cells as described. From day 3 post cell inoculation, mice were injected with rmIL-1β (20 μg/kg) and/or rmIL-23 (20 μg/kg) with or without anti-murine IL-22 (5 mg/kg) thrice weekly for 3 weeks. (**A**) Tumor size was measured continuously. On day 24, tumors were collected for morphological (**B**) and histological analyses (**C**). Histological analyses were performed by H&E staining and Ki-67 immunohistochemical staining. Scale bar: 500 μm. (**D**) Percent of Ki-67-positive cells were counted (n=4). Data are from two independent experiments. Data are mean ± SD, compared using one-way ANOVA test. *p < 0.05.

**Figure 5:**
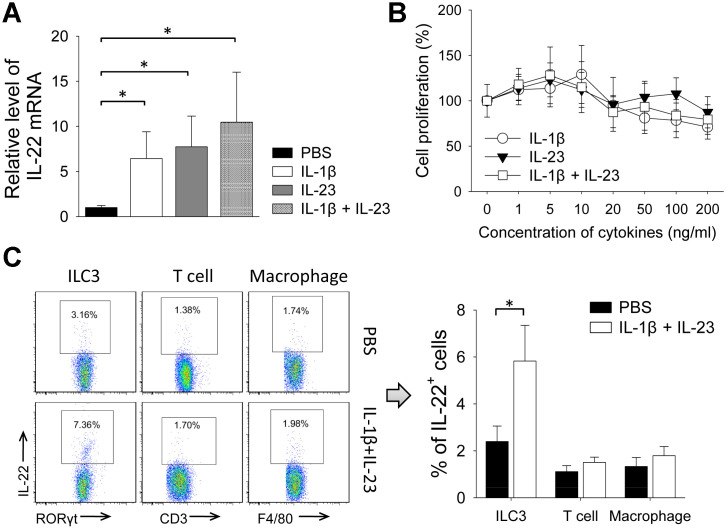
**IL-1β and IL-23 increased levels of IL-22 and IL-22-producing ILC3s in 4T1 tumor.** (**A**, **B**) In 4T1 model, tumors were collected from mice treated by rmIL-1β (20 μg/kg) and/or rmIL-23 (20 μg/kg) on day 21. (**A**) Total RNAs were extracted from tumor tissues and qPCR was applied to analyze levels of IL-22 mRNA (n=4). Data represent fold changes. (**B**) Flow cytometry was applied to analyze IL-22-positive cells in ILC3, macrophage and T-cell (n=4). (**C**) 4T1 cells were cultured with different doses of rmIL-1β and/or rmIL-23 for 24 h. Cells cultured without cytokines were used as control. Cell proliferation was measured with CCK-8 assay (n=6). Data are mean ± SD, compared using unpaired *t* test or one-way ANOVA test. *, *p* < 0.05.

Because IL-1β and IL-23 are upstream cytokines in regulating production of IL-22 [17, 18], we tested the impact of IL-1β and IL-23 on tumor growth. Giving IL-1β or IL-23 increased size of 4T1 tumors. Tumor size was also increased by giving both IL-1β and IL-23, an effect reversed by concurrent use of an IL-22 neutralization antibody (Figure 4A, 4B). IL-1β with or without IL-23 increased percent of Ki-67-positive cells in the 4T1 tumors, which was decreased by concurrent use of the IL-22 antibody (Figure 4C, 4D). Giving IL-1β and/or IL-23 increased IL-22 mRNA levels in the 4T1 tumors (Figure 5A). IL-1β and IL-23 increased percent of IL-22-producing ILC3 in tumor tissue (Figure 5B). However, IL-1β and IL-23 did not show a direct proliferative effect on 4T1 cells *in vitro* (Figure 5C). These results indicate IL-22 might be important in mediating the tumor-promoting effect of IL-1β and IL-23.

## DISCUSSION

In this study, we showed IL-22 exerted a proliferative effect on breast cancer cells in a STAT3-dependent manner. In 4T1 tumor tissue, ILC3 is a major cellular source of IL-22 which can be increased by giving IL-1β and IL-23.

IL-22 plays a role in regulating inflammatory diseases with a predominant anti-inflammatory effect. More recently, IL-22 was reported to be an important mediator of epithelial homeostasis [7]. These characters render a cancer-promoting function of IL-22 in epithelial cancers. This was implicated in the correlations between IL-22 and several types of carcinoma such as colon cancer and hepatocellular carcinoma [11–14]. Some recent studies indicated increased IL-22 level was associated with progression of breast cancer [16, 19]. We showed giving IL-22 increased proliferation of breast cancer cells including estrogen receptor-positive MCF7 cells and triple-negative 4T1 and MDA-MB-231 cells, which indicates IL-22-mediated proliferative effect might not depend on expression of estrogen receptor. Others reported expression of IL-22 in human breast cancer [16, 20]. However, the correlation between IL-22 level and receptor type of tumor cell is unclear in human breast cancer.

In the absence of exogenous IL-22, endogenous IL-22 level was also up-regulated in 4T1 model. Endogenous IL-22 might also exert proliferative effect in breast cancer, which can be confirmed by a test in IL-22 gene knock-out mice. Because MCF7 and MDA-MB-231 xenograft models were established on immune deficient mice, we analyzed IL-22 production in the 4T1 model which was performed with immune competent mice. IL-22 producing cells were enriched in T-cell, macrophage and ILC3, with ILC3 as the major producer. A recent study reported that ILC3 infiltration promoted lymphatic metastasis of breast cancer [15]. On the other hand, exhaustion of T-cell function can be found in breast cancer [21]. IL-22-mediated anti-inflammatory effect might further impair the anti-tumor function of T-cell. These findings suggest IL-22 is a cancer-promoting factor in breast cancer, and blocking IL-22 function might reduce tumor progression. IL-22 antibody was reported to reduce growth of other types of tumors [22, 23]. Nonetheless, results from human studies are needed to confirm the correlation between IL-22 and breast cancer.

Activation of STAT3 pathway was reported in a variety of malignancies including breast cancer [24]. Chronic inflammation was observed in pathogenesis of breast cancer [6]. IL-6 is a strong stimulator of STAT3 pathway which is important in regulating survival and growth of breast cancer cells [25]. We showed IL-22, an IL-10 family member, increased phosphorylation of STAT3 in 4T1, MCF7 and MDA-MB-231 cells. Inhibition of STAT3 reversed the proliferative effect of IL-22 on these cells. Moreover, STAT3 can be activated by other stimulators such as EGF and VEGF [26], which are important in formation of stromal compartment in breast cancer [24].

The role of IL-1β in breast cancer has been reported [27, 28]. Increased level of IL-23 was also observed in human breast cancer [29]. However, it is unclear whether these two cytokines mediate a direct effect on breast cancer cells. We showed IL-1β and IL-23 did not directly increase cell proliferation in cell culture, but rather increased tumor size in the transplant model *in vivo*. Interestingly, this effect was blocked by an IL-22 neutralization antibody. IL-1β and IL-23 increased IL-22 production of ILC3 in the tumor tissue. Thus, a possible mechanism might be IL-1β and IL-23 promoting breast cancer progression *via* IL-22. It has been well established that IL-1β and IL-23 stimulate production of IL-22 in immune cells [17, 18]. Macrophage, a major component of tumor immune microenvironment, is a potent producer of IL-1β and IL-23 [30, 31]. Pathogenic macrophages often present in human breast cancer tissues, and the macrophage-derived factors are involved in tumor progression [6, 32]. It can be deduced that pathogenic macrophages might up-regulate production of IL-22 in ILC3. In addition, we cannot rule out that IL-1β and IL-23 mediate breast cancer progression through immune-regulatory functions. Others reported breast cancer cell-derived IL-1α up-regulated IL-22 production of T cells in an AhR- and RORγt-dependent manner, which indicated a direct crosstalk between cancer cells and immune cells [16].

In conclusion, we confirm the proliferative effect of IL-22 in breast cancer. ILC3 is a primary cellular source of IL-22 in tumor tissue. Our findings suggest blocking IL-22 function might reduce IL-1β- and IL-23-induced tumor progression of breast cancer.

## MATERIALS AND METHODS

### Mice

Female BALB/c mice and BALB/c Nude mice (18-20g) were purchased from Vital River (Charles River, Beijing, China). Mice were bred in a special pathogen free room. Mice were anesthetized by inhaling isoflurane before invasive operations. All procedures regarding animal care and experiments were approved by the Experimental Animal Care and Use Committee of Xuzhou Medical University.

### Cell culture

MCF7 cells (ATCC HTB-22) were cultured in DMEM medium (Thermo Fisher Scientific, Waltham, MA, USA) supplied with 10% FBS (Gibco, Thermo Fisher Scientific). 4T1 (ATCC CRL-2539) cells were cultured in RPMI-1640 medium (Sigma-Aldrich, Shanghai, China) supplied with 10% FBS. MDA-MB-231 (ATCC HTB-26) cells were cultured in Leibovitz's L-15 medium (Thermo Fisher Scientific) containing 10% FBS in an incubator without CO_2_ equilibration.

### Reagents

Recombinant murine IL-22 (rmIL-22), rmIL-1β and anti-murine IL-22 were purchased from PeproTech (Cat# 210-22, Cat# 211-11B and Cat# 500-P223, Rocky Hill, NJ, USA). rmIL-23 was purchased from BioLegend (Cat# 589002, San Diego, CA, USA). Recombinant human IL-22 (rhIL-22) was purchased from STEMCELL Technologies (Cat# 78038, Shanghai, China). Stattic was purchased from ApexBio (Cat# A2224, Houston, TX, USA).

### Tumor models

4T1 cells (2×10^5^) were injected into a single mammary fat pad of BALB/c mice. BALB/c Nude mice were inoculated subcutaneously with 2×10^6^ MCF7 cells or 2×10^6^ MDA-MB-231 cells. Mice were injected intraperitoneally (i.p.) with PBS, recombinant cytokines (20 μg/kg) or anti-murine IL-22 (5 mg/kg) thrice a week for up to 3 weeks after cell inoculation. Tumor size was measured continuously. Tumor volume was calculated as: Tumor volume (mm^3^) = length (mm) × width (mm) × width (mm) × 0.5.

### H&E staining and immunohistochemistry

Tissue slides of tumors were prepared from samples collected from *in vivo* experiments. H&E staining was performed as described previously [33].

To detect Ki-67-expressing cells, immunohistochemistry was performed on tissue slides. Ki-67 antibodies were purchased from ZSGB-BIO (Cat# ZM-0166, Beijing, China) and Affinity Biosciences (Cat# AF0198, Cincinnati, OH, USA). Second antibody was peroxidase-conjugated goat anti-rabbit IgG (ZSGB-BIO, Cat# PV-6000). DAB substrate (ZSGB-BIO) was used for visualization of positive cells.

### Cell proliferation

Cell proliferation was measured with a Cell Counting Kit-8 (CCK-8) from DOJINDO (CK04, Tokyo, Japan). Cells were cultured with CCK-8 reagent for 1 hour followed by detection of optical density at 450 nm.

### Western blotting

Proteins were extracted from cells using Cell Lysis Buffer (Cell Signaling Technology, Danvers, MA, USA). Western blot analysis was performed to detect protein levels with the following antibodies: Phospho-STAT3 (Tyr705), STAT3 (4904), Phospho-STAT5 (Tyr694), STAT5 (94205), Phospho-ERK (T202/Y204), ERK (4695) and GAPDH (D16H11) from Cell Signaling Technology.

### Quantitative polymerase chain reaction

Quantitative polymerase chain reaction (qPCR) was performed to detect IL-22 mRNA as described previously [34]. RNA was isolated using TRIzol (Invitrogen, Thermo Fisher Scientific). cDNA was synthesized using PrimeScript cDNA Synthesis Kit (Takara Bio Inc., Shiga, Japan). cDNA concentrations were analyzed by qPCR with LightCycler 480 SYBR Green I Master qRT-PCR kit (Roche, Mannheim, Germany). GAPDH mRNA level was used for normalization. qPCR was performed on an LC480 cycler (Roche) with the following primers (Thermo Fisher Scientific, Shanghai, China): IL-22 (TCGCCTTGATCTCTCCACTC and GCTCAGCTCCTGTCACATCA) and GAPDH (TTGATGGCAACAATCTCCAC and CGTCCCGTAGACAAAATGGT). Relative mRNA levels are expressed as fold change calculated from –ΔΔC_T_ values.

### Isolation of mononuclear cells from tissues

To isolate mononuclear cells from lymph node, samples were mechanically dissociated in RPMI-1640 medium containing 2% FBS. Cells were recovered by centrifugation in Lymphocyte Separation Medium (DAKEWE, Shanghai, China).

To isolate mononuclear cells from small intestine, samples were obtained and Peyer’s patches were removed. Samples were cut into small pieces and incubated with 5 mM EDTA, followed by a digestion with collagenase type I (1 mg/ml), collagenase type IV (0.5 mg/ml) and DNase I (1 mg/ml). Remained tissues were digested with dispase (0.5 mg/ml) and collagenase type I (1 mg/ml). Released cells were collected from every digestion. Cells were recovered by centrifugation in Percoll buffer (GE Healthcare Bio-Sciences, Pittsburgh, PA, USA).

To isolate mononuclear cells from tumor, samples were twice digested with collagenase type I (1 mg/ml), collagenase type IV (0.5 mg/ml) and DNase I (1 mg/ml). Released cells were centrifuged in Percoll buffer. Enzymes were purchased from Sigma-Aldrich (Shanghai, China).

### Flow cytometry

For analyzing IL-22-producing cells, isolated mononuclear cells were stimulated with PMA (Sigma-Aldrich), ionomycin (Sigma-Aldrich) and Brefeldin A (Thermo Fisher Scientific) for 4 h, followed by treatment with Transcription Factor Staining Buffer (eBioscience, Thermo Fisher Scientific). Cells were stained with the following antibodies: anti-CD45 (30-F11), anti-IL-22 (Poly5164), anti-CD3 (145-2C11), anti-CD4 (RM4-5), anti-F4/80 (T45-2342), anti-RORγt (Q31-378) and anti-IL-7Rα (SB/199). T cells are defined as CD3^+^. Macrophages are defined as F4/80^+^. CD3^-^CD4^+^IL-7Rα^+^RORγt^+^ cells were considered as ILC3. Antibodies were from BioLegend (San Diego, CA, USA), eBioscience (Thermo Fisher Scientific) or BD Biosciences (San Jose, CA, USA). Cells were acquired and analyzed on an LSRFortessa flow cytometer (BD Biosciences).

### Statistics

Group size (n) refers to independent values. Data are presented as mean ± standard deviation (SD). Comparison of means was performed with unpaired Student *t* test or one-way ANOVA test followed by Bonferroni correction. P-value < 0.05 was considered statistically significant.
